# “His brain works in a different way”: siblings’ understanding of autism

**DOI:** 10.3389/fpsyt.2025.1506057

**Published:** 2025-02-13

**Authors:** Krister W. Fjermestad, Nora Hjelde Lervik

**Affiliations:** ^1^ Department of Psychology, University of Oslo, Oslo, Norway; ^2^ Frambu Resource Centre for Rare Disorders, Siggerud, Norway

**Keywords:** siblings, knowledge, qualitative research, children, autism

## Abstract

Siblings of autistic children are at increased risk of mental health problems. Lack of autism knowledge may contribute to this risk. We examined siblings’ autism knowledge using the Sibling Knowledge Interview (SKI) conducted by clinicians before a sibling intervention. The sample comprised 28 siblings (61% boys; 39% girls) aged 8 to 12 years. All had a brother or sister with a clinically confirmed autism diagnosis. Audiotaped recordings of the interviews were transcribed and analyzed thematically. We identified six main themes: (1) definition, including diagnostic label, localization, explanation, and etiology, (2) regulation-, behavior-, social-, and cognition-related challenges, (3) strengths, (4) health service and family-based interventions, (5) misconceptions, and (6) lack of knowledge. Siblings displayed some knowledge about the key aspects of autism but also expressed insecurity, lack of knowledge, confusion, and misconceptions. Siblings displayed a narrow vocabulary to describe their brother’s or sister’s autism diagnosis. Few siblings provided elaborate and rich answers. Several siblings provided vague descriptions. The youngest siblings had particularly few verbally rich answers and displayed limited knowledge. The findings indicate that siblings need more knowledge about their brother’s or sister’s autism diagnosis. Research is needed on how this information should be provided.

## “His brain works in a different way”: siblings’ understanding of autism

When a child is autistic, multiple challenges linked to this neurodiversity, such as anxiety, communication difficulties, rigidity, and behavior problems may strain the child’s family system (1). Reviews have demonstrated negative socio-emotional outcomes also for siblings of autistic children (2, 3). The risks include more anxiety, depression, and behavior problems, and lower self-esteem (3). Furthermore, studies have shown that siblings of autistic children have poorer peer relationships, less peer involvement, and poorer academic functioning compared to other children (4). Importantly, positive outcomes for siblings have also been reported, and studies have identified prosocial behavior, social support, and perspective taking as particular strengths in siblings of children with neurodevelopmental disorders, including autism (5–7).

A recent review of studies of siblings of autistic children identified multiple positive outcomes for siblings but also found lack of autism knowledge to be a key challenge for siblings (1). The focus of the current report is therefore on autism knowledge in siblings. Siblings’ experience of emotional distress may partially be associated with lack of autism knowledge (1). Examining and potentially enhancing knowledge may also be key to building strength and empowering siblings. In the absence of information, siblings tend to make their own guesses (8). This may result in ideas that contradict medical facts. Empirically based examples include believing that a disorder may disappear by itself or suggesting treatment procedures that are not indicated (e.g. brain surgery; 9). Importantly, knowledge about a diagnosis may promote positive effects or resilience in siblings (10, 11). Mapping siblings’ knowledge about the diagnosis of the autistic child may enable healthcare personnel and parents to better accommodate siblings’ needs. It may be particularly useful to examine siblings’ perspectives qualitatively, to identify which words and labels siblings use for autism, and how they understand this. Such information can guide the information health care providers give to siblings and may also inform what questions health personnel should ask siblings.

A few studies have examined autism knowledge among siblings. In a study of 63 siblings aged 5 to 17 years of autistic children, Glasberg (12) found siblings often displayed lack of knowledge about autism, but that their knowledge and understanding increased with age. In a study of an 8-week support group intervention for 26 siblings of autistic children or related disorders aged 6 to 16 years; Smith and Perry (13) found a significant increase in siblings’ autism knowledge from pre- to post-intervention measured with the 20-item Autism Knowledge Measure for Young Children. In a controlled study in which 22 siblings aged 6 to 15 years of autistic children received a psychoeducational intervention, Brouzos et al. (14) found a significant increase on a “Knowledge of Autism” measure for the psychoeducational group but not for controls. Less understanding with lower age indicates that it is important to consider autism-specific sibling knowledge among younger siblings.

Other studies have considered sibling knowledge using the Sibling Knowledge Interview (15). The Sibling Knowledge Interview is a structured interview to assess siblings’ knowledge about a disorder on the dimensions name of the disorder, core symptoms, function, cause, and treatment (15). The SKI is not developed for a specific disorder but has been used in samples that included siblings of autistic children. In a 6-week sibling support intervention study in which 12 (of 54) siblings aged 8 to 13 years had an autistic brother or sister, Lobato and Kao (15) found a significant increase in disorder knowledge from pre- to post intervention. Using the same intervention in study in which 20 siblings (of 54) aged 8 to 12 years had an autistic brother or sister, Granat et al. (16) also found a significant increase in disorder knowledge from pre- to post intervention. In a 5-session sibling and parent intervention study in which 25% of 99 siblings (aged 8 to 16 years) had an autistic brother or sister, Haukeland et al. (17) found a significant increase in sibling disorder knowledge from pre-intervention to 6-months post intervention.

The purpose of the current study is to examine what siblings aged 8 to 12 years know about their brother or sister’s autism. Since the study is qualitative, we did not have pre-generated hypotheses. Rather, we expect the findings to inspire the design of future sibling knowledge studies, including generating testable hypotheses. Recently there has been a shift from a medical deficit focus toward stakeholder-informed and co-produced research (e.g., with autistic researchers and their families) including the personalized needs and strengths of each individual autistic youth (18). In line with this shift, in this study we included young siblings and used the term “autistic youth”, which many users prefer to the traditional “person with autism”.

## Method

### Sample and procedures

The participants are from an ongoing randomized controlled trial (RCT) of a group-based parent-sibling group intervention called SIBS (19). The RCT includes siblings aged 8 to 16 years of children with various neurodevelopmental conditions including autism (20). SIBS was previously positively evaluated in an open trial (i.e., no control group), including increased disorder knowledge from before SIBS to 6-months after SIBS (17). In the current report, we only used pre-intervention data and focus on families of autistic children. The Norwegian Autism Association was involved in preparing the RCT.

The current sample comprised 28 siblings (61% boys, 39% girls; 92% European-White; 0.5% European-African; 0.5% Mixed/Other) aged 8 to 12 years of autistic children. The autism diagnosis was parent-reported and verified against ICD-10 based medical records (21) as F84.5 Asperger syndrome (n = 17), F84.0 childhood autism (n = 5), or F84.1 atypical/non-specified autism (n = 6). In terms of socio-economic background, 67% of mothers and 58% of fathers had >4 years of post-high school education and 79% of parents rated the family economy as good or very good. The participants were recruited through multiple pathways. Some sites invited families in their database, others advertised in waiting rooms. The study was also advertised through autism user associations. The study was approved by the Regional Committee for Medical and Health Research Ethics – South East (#2018/2461). All parents provided informed consent on behalf of the children.

## Measure

The Sibling Knowledge Interview (SKI) (15) is a structured interview to assess siblings’ knowledge about a disorder. The SKI was developed in a sample of children aged 8 to 13 years (15). The interview covers the name of the disorder, core symptoms (i.e., body parts, manifestation, function/impairment, e.g., “what do autistic children have trouble doing?”), cause (e.g., “how did your brother/sister get autism?”, and treatment (e.g., “how does autism get treated?”) (15). The interview was administered in Norwegian by trained clinicians. The interviews lasted 2 to 10 minutes (median 4 minutes).

### Data storage and analysis

The interviews were audio-recorded using an app that automatically transfer the recordings to a data protection-agency approved online storage system for sensitive data. The material was analyzed with thematic analysis (22) in five stages: (1) Dataset familiarization. Here, the second author listened to the audio recordings several times as she transcribed them, while making short notes about potential patterns in the data. (2) Data coding. Here, we systematically went through the dataset extracting segments which appeared interesting. (3) Initial theme generation. Here, we further developed the labels created in phases 1 and 2 by rearranging statements into new categories in clusters. (4) Theme development and review. Here, we went back to the original dataset in light of the themes, included new statements and kept, removed, or changed themes. (5) Theme refining, defining, and naming. Here, we refined and finetuned and named themes based on reflections and discussion.

## Results

**Figure 1:**
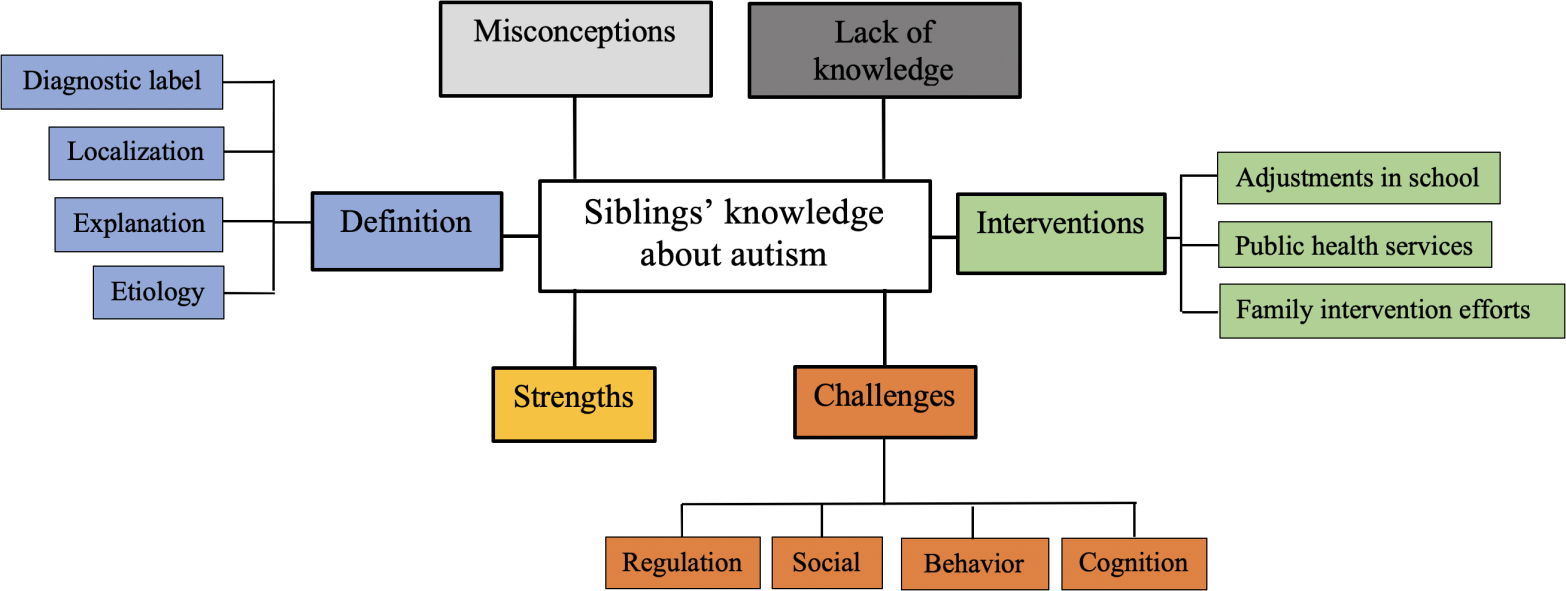
Sibling knowledge themes identified among 28 siblings of autistic children.

We identified six main themes (See **Figure 1**).

### Definition

Definitional issues concerned diagnostic label, localization, explanation, and etiology. Most siblings (79%) used the term “autism” or “Asperger syndrome”. In terms of location, most siblings answered the brain (n=12) or the head (n=7). Relating to various aspects of “difference” was common when asked to explain autism (*“It is not really a disease, it is just that his brain works in a different way”*). In terms of etiology, 12 siblings (43%) said autism is congenital (*“He was born with it”*).

### Challenges

The siblings described four main challenges. First, *regulation challenges* concerned emotion regulation, fluctuating energy levels including tiredness and increased sleep needs, and the ability to stop and/or switch activities (“*A disease that sort of makes it so that you can’t control yourself”*). Second, *social challenges* concerned communication, social competence, and withdrawal from social interaction (“*They struggle with being with a lot of people, so my brother* sp*ends much time in his room, he basically never comes out”*). Third, *behavior challenges* concerned special interests, violence, compulsions, or struggling with activities of daily living (“*You get one* sp*ecial thing you like to look at or to do”;* “*Sometimes he hits mum; after, when he gets that it’s very wrong, he says that he is mean and then he hits himself”*). Fourth, *cognition challenges* concerned delayed development, thought processes, concentration, and sensory sensitivity (“*One can talk loudly, but one must not do it around my brother because he hears very well”*).

### Strengths

Several siblings mentioned autism-related strengths, including physical abilities, general knowledge abilities, school subjects, memory, personal skills, gaming, and creativity strengths (“*My little brother, he remembers very well”*).

### Interventions

Siblings mentioned autism intervention like adjustments in school, public health services, and efforts by family members. The specific school adjustments described included being homeschooled, having fewer lessons, having an additional teacher, having additional breaks, and being allowed to use an iPad. In terms of the public health system, the only service specified was Child and Adolescent Mental Health Clinics. Family interventions included being patient, staying calm, letting the autistic child be by themselves, breaking down tasks step by step, doing things slower, having a companion to help and assist the autistic child, and trying to make the autistic child interested in being social.

### Misconceptions

Sibling statements that reflected misconceptions and confusion concerned autism-related terms (“*An abbreviated word* [for autism] *is ADD*”), diagnostics (“*I guess that there is a test where they connect electrodes or something to the head that measure brain frequency or something”)*, symptoms (“*His hair grows slower*”), localization (“*Mouth”*); and treatment (“*Maybe it will go away by itself”*).

### Lack of knowledge

Siblings displayed lack of knowledge explicitly by giving no verbal answer, or implicitly by displaying misconceptions. Thirteen siblings (46%) displayed lack of knowledge in terms of etiology. Ten siblings (36%) displayed lack of knowledge regarding manifestations. Nine siblings (32%) displayed lack of knowledge regarding explanations, challenges, and treatment, respectively. Seven siblings (25%) displayed lack of knowledge regarding localization and strengths, respectively. Six siblings (21%) showed a lack of knowledge about the diagnostic label. Examples of the statements coded as lack of knowledge include *“I do not remember”, “I have no idea how to know”, and “No one has told me how to find out or anything”*.

### Quality reflections on siblings’ statements

There was much variation in how verbally active the siblings were, and answers ranged from simple “*I don’t know*” to lengthy explanations. Most siblings gave short, non-elaborative answers. The statements represent a continuum from vaguer statements like “*One tries to help them to understand different things”* to more specific advice like “*Breaks, like in the middle of class and stuff, if she needs it”.* There was also a distinction between confirmatory statements (“*It’s his brain*”) to more tentative answers (*“Maybe the brain?”*).

The 8-year-olds differed qualitatively by having few verbal answers and giving little information about their sibling’s condition. There were no obvious differences between ages 9 to 12 years. This was investigated by comparing interview length in time and by reading the interviews to see if there were qualitative differences between sibling answers.

## Discussion

We analyzed what siblings of autistic children knew about autism based on a structured interview. Across the themes definition, challenges, strengths, and interventions, we found that siblings displayed some knowledge about key aspects of autism but also expressed insecurity, lack of knowledge, confusion, and misconceptions. In their original SKI trial, Lobato and Kao (15) found that siblings of autistic children (as well as intellectual disability and/or psychiatric disorders) explained the disorder less accurately than siblings of children with physical disability or medical disorders. In sample of siblings of autistic children, as well as children with physical/intellectual disability or ADHD, Granat et al. (16) did not find differences in disorder knowledge between the diagnostic groups. However, like us, they found that many siblings (56%) gave vague answers or said “don’t know” regarding the diagnostic label in the baseline SKI assessment Using the SKI, both Lobato and Kao (15) Granat et al. (16), and Haukeland et al. (17) found considerable variation in siblings’ disorder knowledge, and that siblings’ disorder knowledge increased after siblings received interventions that (partly) focused on the disorder. Hence, previous and current SKI findings indicate considerable variation in disorder knowledge among siblings, with lack of knowledge and misconceptions being common. The importance of addressing lack of knowledge among siblings of autistic children was also identified as a key challenge in a recent review (1).

It is also important to consider our findings in light of other studies that have focused on siblings’ autism knowledge but used other measures than the SKI. The percentage of siblings who correctly identified an autism-related term was similar to the findings in Glasberg (12; both 79%). This is interesting since Glasberg (12) included children up to 17 years and the current sample was younger at 8-12 years old. In a sample of siblings of autistic children, Brouzos et al. (14) found large variation in sibling disorder knowledge at baseline, and a significant increase in autism knowledge for an intervention group with no change in the control group. Like the SKI studies, this indicates intervention needs regarding autism knowledge for siblings. In a study of >1000 middle-school children’s knowledge of autism (i.e., not a sibling-focused study), Campbell and Barger (23) found that 46% knew of autism. In light of this, it can be concerning that 21% of siblings of autistic children appear unaware of a diagnostic label for autism. Perhaps, compared to 13 years ago, families are less diagnosis-focused in light of recent attitudes promoting neurodiversity and avoiding problem-focused labels.

Our findings showed that most siblings were aware of key autism features. However, they displayed several misunderstandings, which is in line with previous sibling studies (e.g., 9). In their qualitative study of siblings of children with rare disorders, many of which entails autism features (e.g., Angelman syndrome, 22q11.2 deletion syndrome, Smith Magenis syndrome), Vatne et al. (9) found that many siblings displayed misunderstandings and said they lacked information about the disorder. This corresponds to the answers of several siblings in the current study, who said that they had not been given information about autism. The misunderstandings are likely to be a result of a combination of the sample’s young age and the fact that people tend to view neurodevelopmental disorders, including autism, as complex, abstract, or mysterious (9, 24).

Since there is limited knowledge about autism etiology in the scientific community, it is reasonable to assume that there is little knowledge among siblings as well. Lack of knowledge can lead to misconceptions such as autism might be someone’s fault, or contagious (25). Parents have reported that they often wait to inform children about their sibling’s diagnosis (26). Some siblings have questions about the diagnosis of their brother or sister but may keep them to themselves (8, 25, 26). In a study with 108 siblings aged 0-36 years, Tanaka et al. (8) found that only 67% had been informed the autism diagnosis. Parents informed older siblings significantly more than younger siblings. Many siblings (60%) had asked about the diagnosis before their parents had given an explanation. Furthermore, most siblings had noticed something before their parents explained autism to them (71%) and some had already guessed the diagnosis (7%).

It is important to consider our findings in light of the fact that the siblings were asked focused, specific questions rather than more open, exploratory questions. Open-ended questions typically give more detailed information from children (27). The findings also need to be considered in light of the participants’ age. Younger children are less able to make sense of unfamiliar experiences, have a more restricted vocabulary, and are less used to talking about past experiences than older children (28). The current study has other limitations, such as the SKI was designed to provide specific information about siblings’ knowledge about their brother’s or sister’s diagnosis, and not for deeper qualitative examination. Siblings of children with Asperger syndrome, a diagnostic category that has mostly now been abandoned, were overrepresented in comparison with the other clinical subcategories of autism. Future studies should explore siblings’ knowledge using larger samples and more open-ended qualitative interviews.

The current study has clinical implications. Importantly, siblings’ disorder knowledge should be assessed. Autism knowledge can be assessed using autism specific interviews (e.g. 14) or by transdiagnostic general disorder knowledge interviews such as the SKI (15). We do not have data to support one of these approaches over the other, so choice of measure should depend on clinicians’ preferences and availability of the scales. Relevant for the practice field, these diagnostic interviews are brief and easy to administer and do not require special training. Albeit the scoring systems in research require multiple raters for reliability measures, administration in clinical practice does not need to be complicated.

Attention is also needed about how siblings should best be informed about autism. A few intervention studies have shown increases in siblings’ autism knowledge after intervention (e.g. 14, 17). The intervention components that consider disorder knowledge varies from providing information, using psychoeducation, to open parent-guided exploration of siblings’ knowledge (e.g., 17). The field lacks knowledge about what is the optimal approach. Future studies should address predictors of siblings’ disorder knowledge and take a user-informed approach (including siblings’ and parents’ voices) to identify better ways to enhance siblings’ autism knowledge.

## Data Availability

The raw data supporting the conclusions of this article will be made available by the authors, without undue reservation.
